# Pneumonia Mortality Trends in Children under 5 Years of Age in the Context of Pneumococcal Conjugate Vaccination in Peru, 2003–2017

**DOI:** 10.3390/vaccines11111715

**Published:** 2023-11-14

**Authors:** Carlos A. Sanchez, Michelle Lozada-Urbano, Pablo Best-Bandenay

**Affiliations:** 1 Facultad de Ciencias de la Salud, Universidad Peruana de Ciencias Aplicadas, Lima 15067, Peru; 2 Facultad de Salud Pública y Administración, Universidad Peruana Cayetano Heredia, Lima 15102, Peru; pbestb@gmail.com; 3 Centro Sudamericano de Educación e Investigación en Salud Pública, Universidad Norbert Wiener, Lima 15046, Peru; oriana.rivera@uwiener.edu.pe

**Keywords:** pneumonia, *Streptococcus pneumoniae*, pneumococcal conjugate vaccine, herd immunity, Peru

## Abstract

Worldwide, conjugated pneumococcal vaccines (PCVs) have proven effective against invasive pneumococcal disease, but non-invasive pneumonia is a major cause of mortality in young children and serotypes vary geographically, affecting effectiveness. We analyze nationwide death certificate data between 2003–2017 to assess the impact of PCVs on pneumonia mortality among young children from Peru. We report descriptive statistics and perform timeseries analysis on annual mortality rates (AMRs) and monthly frequencies of pneumonia deaths. Children under 5 years of age accounted for 6.2% (n = 10,408) of all pneumonia deaths (N = 166,844), and 32.3% (n = 3363) were children between 1–4 years of age, of which 95.1% did not report pneumonia etiology. Comparing periods before and after PCV introduction in 2009, mean AMRs dropped 13.5% and 26.0% for children between 1–4 years of age (toddlers/preschoolers), and children under 1 year of age (infants), respectively. A moderate correlation (Spearman’s r = 0.546, *p* < 0.01) in the monthly frequency of pneumonia deaths was estimated between both age groups. Quadratic regression suggests a change in direction around 2005 (highest pneumonia mortality) for both age groups, but percentage change analysis identified an inflection point in 2013 for infants only, not for toddlers/preschoolers, suggesting that the impact of PCVs might be different for each age group.

## 1. Introduction

Worldwide pneumococcus is reported as a significant cause of community-acquired pneumonia in both pediatric and adult populations. According to the World Health Organization (WHO), pneumonia accounts for 14% of all deaths in children under 5 years of age [1], which could become a substantial public health problem in developing countries with a significantly younger population [2]. In addition to causing respiratory infections like pneumonia, *Streptococcus pneumoniae* (pneumococcus) also causes invasive infections like sepsis and meningitis [3], which are more severe but less common. Pneumococcal conjugate vaccines (PCVs) are important public health tools that are promoted by the WHO and incorporated into national immunization schemes all over the world. PCVs are developed using serotypes that are known to cause invasive pneumococcal disease (IPD) in children in high income countries, with varying impact on non-invasive disease elsewhere (i.e., most pneumonia cases are non-invasive) [2,4]. A review of six randomized controlled trials in five countries on PCV efficacy among children less than two years of age reported an 80% (95% confidence interval [CI] 58–90%, *p* < 0.0001) impact in IPDs caused by vaccine-serotypes, 58% (95% CI 29–75%, *p* = 0.001) for IPDs caused by all serotypes, 27% (95% CI 15–36%, *p* < 0.0001) for pneumonia defined by X-ray, and 6% (95% CI 2–9%, *p* = 0.0006) for clinically defined pneumonia [5]. Similarly, a systematic review among children under 5 years of age in Latin America (including Peru) reported that PCV effectiveness was higher in hospitalizations due to X-ray confirmed pneumonia (range: 8.8–37.8%) than in clinically diagnosed pneumonia (range: 7.4–20.6%) [6]. Thus, studies show there is little or no protection from PCVs against the less specific (but very common) endpoint of clinical pneumonia [2], which excludes any specific etiology, usually in most developing countries that lack laboratory confirmation and rely routinely on syndromic diagnosis and treatment. The low efficacy of PCVs with non-invasive pneumonia is commonly overlooked because of the tremendous potential impact given the high burden of pneumonia in infants [7], but this only holds true if pneumococcus is indeed a major cause of pneumonia, which cannot be confirmed in every location or setting. The distribution of pneumococcus serotypes in Latin America varies within the region, and the coverage from vaccines that incorporate specific serotypes also varies in different parts of the world [8]. The distribution of serotypes that cause disease can also vary over time and by age, disease syndrome, disease severity, geographical region and the presence of antimicrobial-resistant genes [9,10]. Estimating PCV efficacy against confirmed pneumococcal pneumonia is constrained by limitations establishing etiology in cases of non-bacteremic (non-invasive) pneumonia [2]. To ensure continued adequate vaccine coverage in countries like Peru, laboratory surveillance is critical to monitor circulating pneumococcus serotypes.

Vaccination programs have variable and sometimes unexpected effects in different countries since multiple factors play a role in determining pneumococcal disease evolution, such as natural competition, cross immunity or antibiotic exposure. For example, the first 7-valent PCV (PCV7) included the serotypes that represented approximately 86% of pediatric invasive strains of *S. pneumoniae* in the United States [2]. PCV7 successfully lowered rates of invasive disease in Germany, Switzerland and the United Kingdom (introduced PCV in 2006) but not in France (introduced PCV in 2003). By assessing serotype distribution, French researchers were able to determine that after the introduction of PCV7, there were indeed fewer meningitis cases due to PCV7-targeted strains, but no overall decrease due to serotype replacement (non-vaccine serotypes become more prevalent). In 2010, the 13-valent PCV13 replaced PCV7, but bacterial meningitis cases only began to decrease in 2014 [11]. Thus, simply adding more serotypes does not necessarily guarantee an effective immunization program. A new 20-valent PCV, which was originally approved for IPD and pneumonia in adults [12], has recently been approved for infants and children in the United States [13]. The additional seven serotypes are reported to be among the most common serotypes causing pediatric IPD in countries like the United States, but this might not automatically extrapolate to non-invasive pneumonia in developing countries.

Before its introduction in the Peruvian national immunization program, PCVs were reported by the WHO as a major advance in the prevention of diseases caused by pneumococcus, including both invasive infection and pneumonia with bacteremia [2]. More recently, WHO reports that “Both PCV10 and PCV13 have substantial impacts against pneumonia, vaccine-type IPD and nasopharyngeal (NP) carriage caused by the respective vaccine serotypes” [9]. A recent study in the United States is far more optimistic [10]. Consequently, PCVs are listed in the Peruvian national immunization program as protection against pneumonia, meningitis and otitis media [14], even though the serotype causing the pneumonia is usually unknown. We believe the potential of PCVs to prevent non-invasive pneumonia in children in other parts of the world should be fully tested before implementing international immunization programs. The effectiveness of PCVs protecting against hospitalizations and mortality needs to be evaluated, but different health outcomes usually require different study designs, depending on available data sources. Due to the lack of laboratory confirmation, most studies attempt to measure the overall effectiveness of the vaccine in preventing radiologically defined pneumonia, irrespective of etiology [2]. To our knowledge, few published studies have looked critically to the benefits of PCVs on childhood pneumonia mortality at the population level in developing countries. The main aim of this study is to assess the impact of PCVs in pneumococcal pneumonia mortality of children under 5 years of age in Peru, a middle-income country in South America that has included PCVs in their national immunization programs for the last 15 years.

## 2. Materials and Methods

We conducted a retrospective, observational study analyzing longitudinal data for child pneumonia mortality between 2003 and 2017 to estimate and model mortality trends at the national level in Peru using timeseries analysis to compare the periods before and after the introduction of pneumococcal conjugate vaccines.

We performed secondary analysis on a governmental healthcare administrative dataset to evaluate any change in trend for pneumonia mortality in the high-risk children group in Peru. Vital statistics datasets from the Peruvian Ministry of Health are considered public domain and are available upon official request. The system captures nationwide information from approximately 120,000 death certificates per year in hardcopy that is later digitalized. The year 2017 is the last year with comparable data since online reporting was fully implemented in 2018.

Population: Potential cases were identified by the “underlying cause of death” in the death certificate using all categories for pneumonia (J12 to J18) in the 10th revision of the International Classification of Diseases (ICD-10). Death certificates almost exclusively reported pneumonia “due to unspecified organism” (J18), so the other pneumonia categories (J12 to J17) were considered outliers and were discarded from this analysis. The final case definition included children under 5 years of age (high-risk age group) who died in Peru during the study period due to all-cause (unspecified etiology) pneumonia, which is our sample population. Most immunization programs include a three-dose schedule during the first year of life, making children under 1 year of age (hereafter “infants”) their target population and a specific age group of interest. Besides infants, the other component of the under 5 years of age high-risk group are toddlers (1–3 years) and preschoolers (3–5 years). This age group of children between 1 and 4 years of age (hereafter “toddlers and preschoolers”) is also expected to benefit from the implementation of PCVs.

Descriptive statistics at the national level included monthly pneumonia death frequencies and annual pneumonia mortality rates (per 100,000 population) estimated using 2009 demographic projections to standardize the effect of population growth during the study period [15]. Rates were not calculated at the monthly scale due to a lack of reliable monthly population estimates. Spearman’s rank correlation test is a non-parametric test (does not assume a normal distribution) used to measure the degree of association between two variables. Correlation is a very basic low-level statistical association and does not imply causation. Linear and quadratic regression models were plotted to visualize the best fit models. All statistical analyses were conducted using the SPSS Statistical Software v23.

We used joinpoint regression to analyze trends in pneumonia mortality rates in order to identify the best fit for inflexion points (“joinpoints”). The significance tests use a Monte Carlo permutation method. The joinpoint trend analysis software is developed by the Surveillance Research Program of the National Cancer Institute Version 5.0.2 (Statistical Research and Applications Branch, National Cancer Institute, Bethesda, MD, USA).

Ethical considerations were minimal, we performed secondary analysis on publicly available datasets without personal identifiers.

## 3. Results

Pneumonia as the underlying cause of death (ICD-10 codes J12 through J18 in), was reported in 11.7% (166,844/1,420,631) of all deaths in Peru during 2003–2017, 14.8% (3363/22,666) of all deaths among toddlers and preschoolers and 9.4% (7394/78,994) of all deaths among infants. Among 166,844 all-cause pneumonia deaths in Peru, 75.6% (n = 126,215) were older adults over 65 years of age and 6.4% (n = 10,757) were young children under 5 years of age, of which 31.3% (n = 3363) were toddlers/preschoolers and 68.7% (n = 7394) were infants. The underlying cause of death was reported as “pneumonia due to an unspecified organism” (ICD-10 code J18) in 95.3% and 95.1% of pneumonia deaths among infants and toddlers/preschoolers, respectively (Table 1).

**Figure 1:**
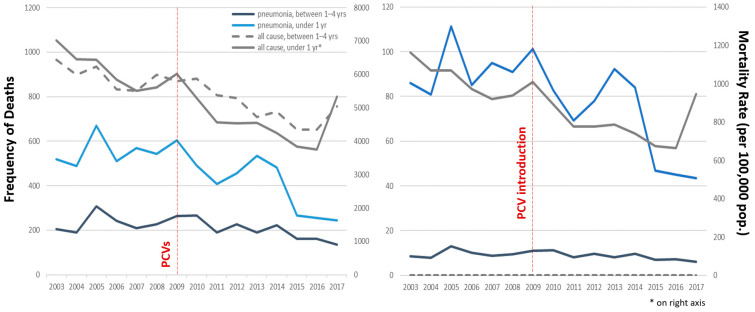
Annual pneumonia and all-cause mortality, frequency and rates in infants and young children, Peru 2003–2017.

All-cause (unspecified) pneumonia mortality in children under 5 years of age is predominantly composed of infants (68.8%, ranging between 61.2% and 73.9% in any given year), who also had the highest number of pneumonia deaths and pneumonia mortality rates, compared to toddlers/preschoolers (maximum pneumonia annual mortality rates 111.3 and 12.9 deaths per 100,000 population, respectively, both in the year 2005). There is a negative (decreasing) secular trend for both frequency of deaths and mortality rates due to pneumonia among young children during the study period (Figure 1). Comparing pre- (2003–2008) to post-(2010–2017) PCV periods, average annual pneumonia crude death rates dropped 13.5% for toddlers/preschoolers and 26.1% for infants (from 9.7 to 8.4 and 91.6 to 67.7 deaths per 100,000 population, respectively). Pneumonia deaths in the infant group drop drastically in 2009, the year of PCV introduction, both in frequency and in rate (Figure 1, blue line), but the effect of PCVs on the trend of pneumonia mortality in toddlers and preschoolers was less evident (Figure 1, black line). Pneumonia mortality shares the same general trend of overall (all-cause) deaths and mortality rates (grey lines), except for the last year of the study period, where there was an increase in overall mortality but not in pneumonia mortality.

**Figure 2:**
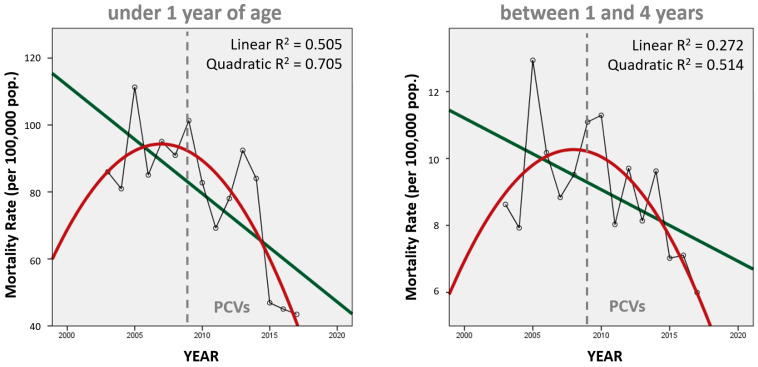
Trends in annual unspecified pneumonia mortality rates in infants and young children, Peru 2003–2017.

Trends in annual mortality are better represented in Figure 2 using a regression line (green) and a quadratic curve (red), which better represent the annual variability in pneumonia mortality rates (according to R^2^ parameters). Both trends fit a curve better than a line (the infant curve perhaps better than the toddler/preschooler curve), which implies a more complex (non-linear) correlation over time. Both curves do suggest that the change from the initial upward trend began before the introduction of PCVs in Peru in 2009, perhaps after 2005, which is the highest value in both graphs.

**Figure 3:**
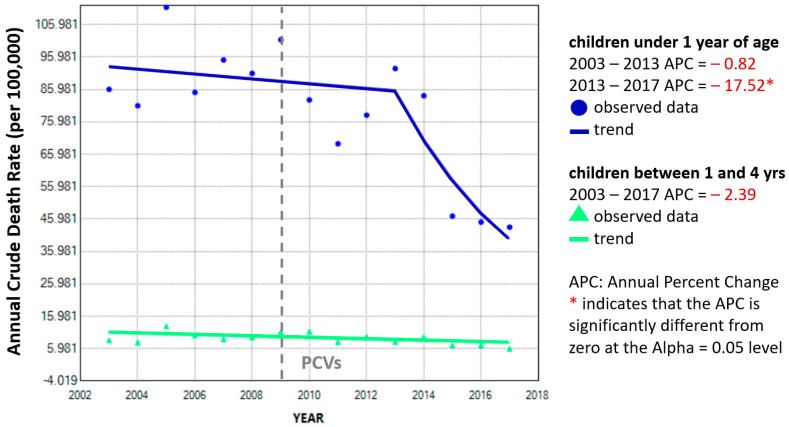
Annual percentage change in mortality rates due to unspecified pneumonia in infants and toddlers/preschoolers, Peru 2003–2017.

The subtle decreasing trend for annual mortality rates among toddlers and preschoolers (green line) seems to be unaffected during the study period (Figure 3). A single best-fit statistically significant inflexion point for annual percent change can be estimated for infant pneumonia mortality rates in the year 2013 (blue line) but not for toddlers/preschoolers (green line).

**Figure 4:**
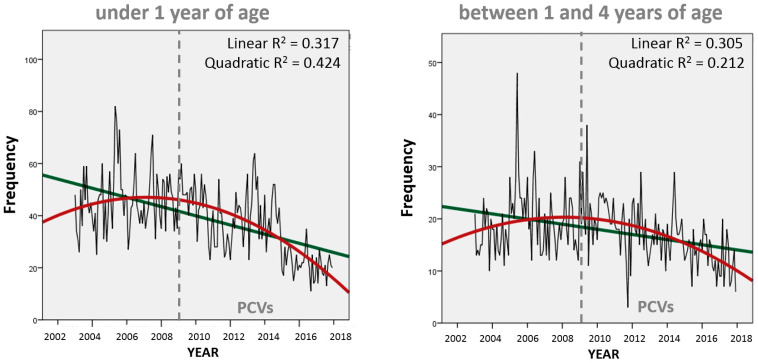
Trends in monthly unspecified pneumonia deaths in infants and toddlers/preschoolers, Peru 2003–2017.

When looking at the frequency of monthly pneumonia deaths, we confirm the initial change in trend direction (around 2005) precedes PCV implementation in Peru for both infants and toddlers/preschoolers (Figure 4) with a final decreasing trend at the end of the study period. At this scale, the quadratic curve (in red) continues to better express the monthly variability for infant deaths (as it did with annual mortality rates) but not for toddlers/preschoolers, which are now better represented (considering R^2^ parameters) through linear regression (in green). Consequently, there is only a moderate (though statistically significant) correlation (Spearman’s r = 0.546, *p* < 0.01) between the frequency of monthly pneumonia deaths in these two groups of young children.

**Figure 5:**
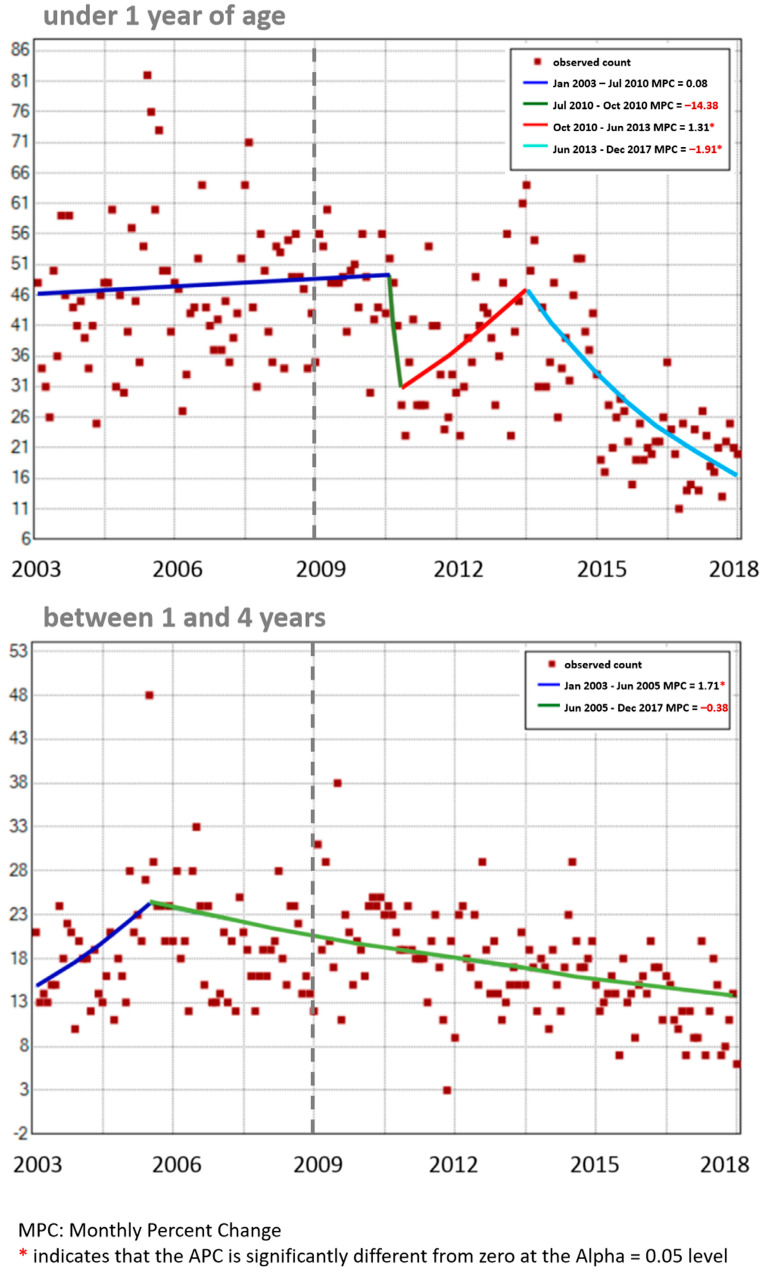
Trends in monthly pneumonia deaths for infants and toddlers/preschoolers in Peru, 2003–2017.

Monthly percentage changes in monthly pneumonia deaths were different for each age group (Figure 5). Among infants (top graph), there was a major (though not statistically significant) drop in the second half of 2010, followed by a statistically significant increase until mid-2013 and a final decrease after that. These results ignore the initial change in trend around 2005 suggested by the quadratic curve in Figure 4 but are consistent with the continuous decrease displayed in Figure 3 and Figure 4 for this age group after 2013. Among toddlers and preschoolers (bottom graph), the single inflection point is estimated to be mid-2005, which agrees with the quadratic curve for this age group seen in Figure 4, and then continues undisturbed by the PCV immunization program.

## 4. Discussion

It must be stated that we do not dispute the demonstrated merits of PCVs in reducing morbidity (i.e., hospitalizations) in countries like the United States [10], but the effect of PCV implementation on mortality in Peru does not seem to follow expected trends. After PCV implementation, the only true decline in pneumonia mortality can be observed between 2013–2017 (Figure 1). This could be explained either by a late response to the initial PCV7 introduced in 2009 or a fast response to PCV10 introduced in 2012. The decline between 2009–2011 arrived to soon to be credited to the vaccine and is actually followed by an increase in pneumonia mortality between 2011–2013, a period with high vaccine coverage. This increase is harder to explain, but it suggests pneumonia mortality may not be directly related to PCVs. Regarding the two young children groups, we can observe that the potential impact of PCVs on pneumonia mortality among infants is debatable while the impact among toddlers/preschoolers is negligible (Figure 1, Figure 3 and Figure 5).

In Peru, before the introduction of PCVs, pneumonia deaths accounted for 20% of all deaths among children under 5 years of age between 1996–2000 [16], but our results during the study period estimate 10.3% and 10.7% before (2003–2008) and after (2010–2017) PCV introduction, respectively. This suggests that the percentage of deaths among young children due to pneumonia had already decreased before PCVs were available and were later relatively unaltered by PCV introduction. There are previous reports on how, in Peru, (1) PCVs alone cannot explain the trend in infant pneumonia mortality (direct benefit to the vaccinated population) [17] and (2) there is a lack of evidence of herd protection (indirect benefit on the older adult unvaccinated population) following PCV introduction [18]. Also, all-cause infant mortality rates have been dropping globally for decades, but in most countries PCVs have only been available relatively recently [19], so other factors may be involved in infant mortality. Our results show that there has been indeed a change in the trend direction for pneumonia mortality among toddlers/preschoolers, but the quadratic curve (Figure 4) and the joinpoint regression (Figure 5) suggest that this change predates the introduction of PCVs in 2009. Compared to infants, the slope of the linear regression is also less steep for toddlers/preschoolers, and their declining trend in pneumonia mortality seems unaffected by the introduction of PCVs in Peru (Figure 3).

The impact of a PCVs may vary for different age groups [20]. In the United States, two years immediately following the introduction of PCV13, a significant decrease in the percentage change (compared to pre-PCV13 levels) in hospital admissions for non-invasive pneumococcal pneumonia was estimated in all age groups except toddlers aged 2–4 years [21]. In any age group, pneumonia mortality trends should always be analyzed within the context of the all-cause mortality trends. In our sample, pneumonia mortality mostly follows the overall decreasing trend of the all-cause mortality both age groups, except for 2017 (Figure 1). Before/after studies (interrupted timeseries) using surveillance data sources were the most common study design used in a systematic review assessing PCV impact and effectiveness in Latin America [6], but such studies (including ours) that limit their comparison to a couple of years before and after PCV introduction may completely miss the underlying secular trend in global infant mortality due to improvements in the quality of life and healthcare [19]. In Peru, pneumonia deaths had already decreased by 45.5% before PCVs became available (between 1996 and 2000) among children under 5 years of age [16]. In Brazil, pneumonia mortality had been steadily decreasing (a 90% reduction between 1980 and 2010) in children younger than 5 years of age before PCV10 introduction in 2010 [22]. Globally, all-cause infant mortality rates have been decreasing since the 1950s [19] and, in those countries that have controlled preventable disease (i.e., infections) in this age group, mortality rates may not decrease much further if most infant deaths are caused by non-preventable diseases (i.e., congenital or genetic), regardless of PCV status. Conversely, a decrease in mortality may be wrongly associated with the vaccine when in fact it could be the anticipated consequence of improvements in education, hygiene, nutrition, and healthcare. In Brazil, only a modest vaccine-associated decline was reported after PCV10 was introduced [22], and no relevant changes in trend for lower respiratory infections were reported after the PCV implementation [23].

Vaccines with broader serotype range are expected to increase vaccine protection [10]. In Peru, the original PCV7 was replaced by PCV10 and later by PCV13 in 2012 and 2015, respectively. The promotion of new PCVs includes the statement that “In the United States, there remains a considerable burden of disease attributed to serotypes not included in currently approved pneumococcal conjugate vaccines” [13], but PCVs themselves may have contributed to the emergence of these replacement serotypes in different countries following pneumococcal immunization programs, preventing the disease from disappearing completely as new non-vaccine serotypes become predominant. In the United States, where PCVs were introduced in the year 2000, although IPD cases in children caused by serotypes contained in the original PCV7 declined through 2005, overall IPD rates leveled off and plateaued in the beginning of 2002 [24]. In France, instead of the expected decrease after PCV7 introduction, there was an increase in meningitis caused by serotypes not covered in the vaccine, and the serotypes emerging in France were different than those emerging in other countries [11]. In the United States, serotype replacement was not reported in clinical trials in which IPD was the endpoint, but sustained increases in the population-based incidence of IPDs caused by serotypes not included in the vaccine were reported by surveillance studies after widespread use of PCV7 [2].

The WHO acknowledges it is difficult to determine the proportion of pneumonia that is due to pneumococcus [9]. Most pneumonia cases do not achieve laboratory confirmation, so the real burden of disease from pneumococcal pneumonia is unclear in many countries and so is the potential benefit of PCVs. This lack of testing for a causal pathogen has also been reported in other Latin American countries [20] and elsewhere in the world [25,26,27]. WHO recommendations suggest that the decision to switch to a vaccine with higher serotype coverage should be based on the local serotype prevalence and the evaluation of the additional benefit of expanding the number of vaccine serotypes [9]. Back in 2007, WHO data supported switching from the 7-valent to the 10-valent vaccine, increasing the proportion of serotypes covered by the vaccine in the United States, Europe, Africa and some parts of Asia, namely from 86% to 88%, 74% to 84%, 67% to 81% and 43% to 66%, respectively, but there were no estimates available for Latin America [28]. Without serotype surveillance, how can the decision to expand vaccine serotypes be made? The WHO recommendations include surveillance to be conducted in selected countries and defined populations with different epidemiological profiles at least 2 years prior to PCV introduction and continue for at least 5 years after introduction [28]. This is especially important for developing countries with limited resources where all new vaccines carry higher costs per unit [29]. Designing a better national vaccine strategy will require more research, and continued surveillance is necessary to monitor disease evolution, verify the appropriate local serotypes for new broader-valent pneumococcal vaccines, and recognize unexpected changes due to unknown internal or external factors [11].

### Limitations

There are potential confounding factors that may restrict our ability to study the relationship between the PCVs and pneumonia mortality. The lack of a precise beginning date at the regional level may have diluted the impact at the national level during the expanding vaccine coverage period. The introduction of PCVs in Peru was carried out in phases for selected populations after 2007, so there is no unique point in time that determines a precise before and after for the whole country. The vaccine was officially available nationwide in 2009, when national vaccine coverage began to be estimated. Observational studies assessing the impact of vaccines ideally require data on the distribution and coverage of vaccines, vaccination schedule and types of vaccine [30]. Vaccine coverage at the local level may affect pneumonia occurrence at the national level. The distribution of PCVs in Peru during the first two years before full implementation in 2009 is unclear, but it might not be relevant since national vaccine coverage rose from 0 to 8.7% during 2009.

Using vital statistics data implies limitations in controlling how data were collected, cleaned, and stored. Data can be underreported and may not be suitable for projection or point estimates, but trends over time might prevail, assuming they are randomly distributed among the study period. We propose that, even with these limitations, if an intervention has a real major impact on the population, that impact should be reflected in data that are systematically collected and periodically reported.

## 5. Conclusions

Our results suggest that other factors may have contributed to the reduced overall child mortality from pneumonia in Peru. Identifying which factors contributed the most to the decrease in young children pneumonia mortality is beyond the scope of the study, but it should be addressed in the future.

Most deaths due to pneumonia are reported without a causal pathogen, so the prevalence of any specific pneumococcus serotype in any specific location remains largely unknown. As additional serotypes are added to new vaccines, questions arise regarding their efficacy in specific settings that were not involved in the design of the vaccine. Considering decreasing global infant mortality trends [19], no evidence of herd immunity [18], an unclear trend in infants [17] and the absence of serotype confirmation in Peru, there is just not enough evidence to support a strong impact from broader PCVs on infant mortality. We can attest to a consistent decrease in pneumonia mortality among young children (infants, toddlers and preschoolers) since 2013 in Peru, but it is unclear whether this trend can be solely explained by PCV intervention. National immunization programs should include impact evaluation in high-risk populations after a vaccine is introduced.

## Figures and Tables

**Table 1:** Frequency of pneumonia deaths for all ages, children under 1 year of age and children between 1 and 4 years of age, according to the ICD-10 code, Peru, 2003–2017.

ICD-10 CATEGORY (Underlying Cause of Death)		All Ages	%	Infants	%	Toddlers & Pre-Schoolers	%
J12	viral pneumonia, not classified elsewhere	1386	0.8	82	1.1	40	1.2
J13	pneumonia due to *Sreptococcus pneumoniae*	58	0.0	1	0.0	1	0.0
J14	pneumonia due to *Haemophilus influenzae*	7	0.0	1	0.0	0	0.0
J15	bacterial pneumonia, not classified elsewhere	7725	4.6	264	3.6	122	3.6
J16	pneumonia due to other microorganisms	84	0.1	1	0.0	3	0.0
J17	pneumonia in diseases classified elsewhere	9	0.0	0	0.0	0	0
J18	pneumonia, due to unspecified organism	157,575	94.4	7045	95.3	3197	95.1
**TOTAL**		**166,844**	100	**7394**	100	**3363**	100

## Data Availability

Data are available upon official request to the MoH.

## References

[1] World Health Organization (WHO) Pneumonia in Children. https://www.who.int/news-room/fact-sheets/detail/pneumonia.

[2] World Health Organization (WHO) (2007). Pneumococcal conjugate vaccine for childhood immunization—WHO position paper-2007. Wkly. Epidemiol. Rec..

[3] Ruvinsky R., Gentile A., Regueira M., Corso A. (2002). Infecciones invasivas por *S. pneumoniae*: Estudio epidemiológico e importancia del desarrollo de un sistema de vigilancia. Arch Argent Pediatr..

[4] Centers for Disease Control and Prevention (CDC) (2022). Pneumococcal Vaccination: What Everyone Should Know. https://www.cdc.gov/vaccines/vpd/pneumo/public/index.html#print.

[5] Lucero M.G., Dulalia V.E., Nillos L.T., Williams G., Parreño R.A.N., Nohynek H., Riley I.D., Makela H. (2009). Pneumococcal conjugate vaccines for preventing vaccine-type invasive pneumococcal disease and X-ray defined pneumonia in children less than two years of age. Cochrane Database Syst. Rev..

[6] de Oliveira L.H., Camacho L.A.B., Coutinho E.S., Martinez-Silveira M.S., Carvalho A.F., Ruiz-Matus C., Toscano C.M. (2016). Impact and Effectiveness of 10 and 13-Valent Pneumococcal Conjugate Vaccines on Hospitalization and Mortality in Children Aged Less than 5 Years in Latin American Countries: A Systematic Review. PLoS ONE.

[7] Pavia M., Bianco A., Nobile C.G., Marinelli P., Angelillo I.F. (2009). Efficacy of pneumococcal vaccination in children younger than 24 months: A meta-analysis. Pediatrics.

[8] Giglio N., Micone P., Gentile A. (2011). The pharmacoeconomics of pneumococcal conjugate vaccines in Latin America. Vaccine.

[9] World Health Organization (WHO) (2019). Pneumococcal conjugate vaccines in infants and children under 5 years of age: WHO position paper. Wkly. Epidemiol. Rec..

[10] Huang L., Wasserman M., Grant L., Farkouh R., Snow V., Arguedas A., Chilson E., Sato R., Perdrizet J. (2022). Burden of pneumococcal disease due to serotypes covered by the 13-valent and new higher-valent pneumococcal conjugate vaccines in the United States. Vaccine.

[11] Alari A., Chaussade H., Domenech De Cellès M., Le Fouler L., Varon E., Opatowski L., Guillemot D., Watier L. (2016). Impact of pneumococcal conjugate vaccines on pneumococcal meningitis cases in France between 2001 and 2014: A time series analysis. BMC Med..

[12] Pfizer (2021). U.S. FDA Approves PREVNAR 20™, Pfizer’s Pneumococcal 20-Valent Conjugate Vaccine for Adults Ages 18 Years or Older. https://www.pfizer.com/news/press-release/press-release-detail/us-fda-approves-prevnar-20tm-pfizers-pneumococcal-20-valent.

[13] Pfizer (2023). U.S. FDA Approves PREVNAR 20^®^, Pfizer’s 20-Valent Pneumococcal Conjugate Vaccine for Infants and Children. https://www.pfizer.com/news/press-release/press-release-detail/us-fda-approves-prevnar-20r-pfizers-20-valent-pneumococcal.

[14] Peruvian Ministry of Health (MoH) (2023). Regular Vaccination Scheme by Life Stages in Peru. https://www.gob.pe/22037-esquema-regular-de-vacunacion-por-etapas-de-vida-en-el-peru%20.

[15] INEI (2009). Perú: Estimaciones y Proyecciones de Población Total, por Años Calendario y Edades Simples, 1950–2050 Lima, Setiembre 2009 Dirección Técnica de Demografía e Indicadores Sociales. Boletín Especial Nº 17.

[16] Huicho L., Trelles M., Gonzales F. (2006). National and sub-national under-five mortality profiles in Peru: A basis for informed policy decisions. BMC Public Health.

[17] Sanchez C.A., Rivera-Lozada O., Lozada-Urbano M., Best P. (2023). Pneumococcal conjugate vaccination and pneumonia mortality trends in children under 1 year of age in Peru, a time series trend analysis, 2003–2017. BMC PH.

[18] Sanchez C.A., Rivera-Lozada O., Lozada-Urbano L., Best P. (2023). Herd immunity in older adults from a middle-income country: A time-series trend analysis of community-acquired pneumonia mortality 2003–2017. Health Sci. Rep..

[19] Sanchez C.A., Rivera-Lozada O., Lozada-Urbano M., Best P. (2023). Infant mortality rates and pneumococcal vaccines: A time-series trend analysis in 194 countries, 1950–2020. BMJ Glob Health.

[20] de Oliveira L.H., Shioda K., Valenzuela M.T., Janusz C.B., Rearte A., Sbarra A.N., Warren J.L., Toscano C.M., Weinberger D.M., Multinational Study for PCV Impact in Mortality Study Team (2021). Declines in Pneumonia Mortality Following the Introduction of Pneumococcal Conjugate Vaccines in Latin American and Caribbean Countries. Clin. Infect. Dis..

[21] Simonsen L., Taylor R.J., Schuck-Paim C., Lustig R., Haber M., Klugman K.P. (2014). Effect of 13-valent pneumococcal conjugate vaccine on admissions to hospital 2 years after its introduction in the USA: A time series analysis. Lancet Respir. Med..

[22] Schuck-Paim C., Taylor R.J., Alonso W.J., Weinberger D.M., Simonsen L. (2019). Effect of pneumococcal conjugate vaccine introduction on childhood pneumonia mortality in Brazil: A retrospective observational study. Lancet Glob. Health.

[23] Camargos P., Nascimento-Carvalho C.M., Teixeira R., França F. (2020). Lower respiratory infections mortality among Brazilians under-five before and after national pneumococcal conjugate vaccine implementation. Vaccine.

[24] Centers for Disease Control and Prevention (CDC) (2008). Invasive Pneumococcal Disease in Children 5 Years after Conjugate Vaccine Introduction-Eight States, 1998–2005. MMWR.

[25] Bartlett J.G. (2004). Decline in microbial studies for patients with pulmonary infections. Clin. Infect. Dis..

[26] Rodgers G.L., Klugman K.P. (2016). Surveillance of the impact of pneumococcal conjugate vaccines in developing countries. Hum Vaccin Immunother..

[27] Aliberti S., Dela Cruz C.S., Sotgiu G., Restrepo M.I. (2019). Pneumonia is a neglected problem: It is now time to act. Lancet Respir Med..

[28] World Health Organization (WHO) (2012). Position paper-2012. Pneumococcal vaccines. Wkly Epidemiol Rec..

[29] Organización Panamericana de la Salud (OPS) Programa Ampliado de Inmunizaciones—Precios de las vacunas para el año 2019. https://www.paho.org/es/file/51814/download?token=jNj5YbeY.

[30] Curcio D., Cané A., Napuri N. (2017). Comment on “Impact of pneumococcal conjugate vaccine in children morbidity and mortality in Peru: Time series analysis”. Vaccine.

